# A dose of doubt: a qualitative study on placebo regulations

**DOI:** 10.3389/fmed.2025.1574022

**Published:** 2025-06-16

**Authors:** Mélina Richard, Manuela Ganz, Bernice S. Elger, Jens Gaab

**Affiliations:** ^1^Institute for Biomedical Ethics, University of Basel, Basel, Switzerland; ^2^Department of Biomedical Engineering, University of Basel, Basel, Switzerland; ^3^Division of Clinical Psychology and Psychotherapy, Faculty of Psychology, University of Basel, Basel, Switzerland; ^4^Center for Legal Medicine, Faculty of Medicine, University of Geneva, Geneva, Switzerland

**Keywords:** placebo use, primary care physicians, clinical decision-making, healthcare regulation, medical ethics

## Abstract

**Introduction:**

Placebo use is common in primary care, yet ethical and legal concerns persist, and few qualitative studies have explored physicians’ views on placebo regulation.

**Methods:**

We conducted semi-structured interviews with 10 primary care physicians from 2 German-speaking Swiss cantons to explore their definitions of placebos, usage in clinical practice, knowledge of existing regulations, and attitudes toward potential regulatory frameworks. Participants were recruited from a publicly available physician registry, yielding a 4.9% response rate.

**Results:**

Participants consistently reported using at least impure placebos in their practice, while references to the use of pure placebos were relatively uncommon. A distinction between pure and impure placebos emerged, with the latter generally viewed as more ethically acceptable. Risk-benefit evaluation was emphasized as the primary justification for placebo use. Most participants had not actively sought legal information, and knowledge about current regulations varied considerably. While clear support for specific regulation was rare, most participants did not perceive it as necessary, often citing distrust in regulatory systems or concerns that formal rules could restrict therapeutic flexibility. Expert bodies such as the Swiss Medical Association were mentioned as potential sources of guidance.

**Discussion:**

The findings highlight a practice-oriented, risk-benefit-driven approach to placebo use, shaped by skepticism toward regulation and limited legal awareness. Despite frequent use, physicians operate in a legally ambiguous space and express limited demand for regulatory clarity, suggesting a need for targeted professional discourse rather than strict formal regulation.

## 1 Introduction

The historical use of placebos in treating ailments is deeply rooted in human culture, possibly dating back to prehistoric times (1). Placebos have been shown to alleviate symptoms such as pain, nausea, fatigue (2), and psychiatric conditions like depression and anxiety (3, 4). However, their use raises ethical concerns about patient autonomy, as withholding information conflicts with patients’ right to informed treatment (5).

Despite these concerns, studies indicate frequent clinical use of both pure (inactive) and impure (non-specific) placebos across various countries (6–8). Reports suggest 29%–97% of general practitioners have used placebos at least once, and 1%–75% use them weekly, with high prevalence linked to impure placebos (7). Discrepancies stem from varying definitions of impure placebos. In previous studies that employed surveys, practitioners viewed placebos as appropriate when benefits outweigh risks and cite motivations like psychological effects, patient expectations, addressing non-specific symptoms, and preventing drug dependency (9–11).

Physicians must disclose the treatment’s rationale, methods, risks, and alternatives to respect patient autonomy (5). The lack of specific legal guidelines has led to calls for clearer regulations (12, 13). Practitioners’ express confusion about placebo rules, with many favoring official guidelines (11, 14). In Switzerland, informed consent is legally required for medical treatments, complicating placebo use, as all interventions without consent could be classified as bodily injury (15). While primary care physicians often face placebo-related dilemmas due to frequent consultations for non-specific symptoms, detailed studies on their views regarding legal regulations are lacking (6). Previous research has relied primarily on quantitative methods, limiting the understanding of physicians’ attitudes and regulatory needs. The rise in placebo research and media coverage, along with evidence supporting open-label placebos (16, 17), highlights the evolving context of placebo use (18).

This study aims to explore the perspectives of Swiss primary care physicians on placebos and related regulations. A qualitative approach will examine their attitudes, knowledge, and needs, providing insights to guide professional bodies and policymakers in developing clearer regulations.

## 2 Materials and methods

This qualitative study explored Swiss primary care physicians’ attitudes toward placebo regulations through semi-structured interviews. Participants shared experiences and opinions regarding placebo use and its legal status in Swiss clinical practice. The semi-structured format allowed follow-up questions for clarification of participants’ statements. These were used when initial responses were ambiguous or required further elaboration. For example, if a participant referenced “regulations,” the interviewer might ask whether they were referring to laws, guidelines, or professional codes. The study included primary care physicians in two German-speaking Swiss cantons, identified via the www.doktorfmh.ch website, and initially contacted via email, with no age restrictions. A follow-up email reminder was sent to non-respondents approximately 2 weeks after the initial contact. Of 491 listed physicians, 205 with available email addresses were contacted in June and July 2022.

### 2.1 Interviews

A semi-structured interview guide was developed based on Helfferich’s “SPSS principle” (German for “collect,” “examine,” “arrange,” and “subsume”) (19), featuring open-ended questions on placebo definitions, clinical use, regulations, and requests (Supplementary Appendix A). Interviews averaged 30 min (range 18–45), and the first interview was used to pilot the interview guide. As the participant responded comprehensively and no issues arose regarding question clarity or structure, only one pilot interview was conducted. To ensure face validity, the guide was discussed within the research group to confirm that the questions covered all relevant content domains. Interviews were conducted at participants’ workplaces from July to September 2022.

### 2.2 Analysis

Using Dresing and Pehl’s (20) transcription criteria, interviews were transcribed verbatim and anonymized to maintain confidentiality. MAXQDA 2022 software was used for transcription and coding. Following Kuckartz and Rädiker’s (21) seven-phase content analysis framework, primary categories were deductively developed based on interview topics, then refined with subcategories derived inductively from the data. Text was coded line by line, grouping meaning units into subcategories or creating new ones when necessary. Categories were reviewed and refined, merging subcategories and adjusting titles to reflect content accurately. The coding system overview is in Supplementary Appendix B. Results are detailed in the following chapters.

## 3 Results

Out of the 205 contacted, 10 primary care physicians (3 female/7 male) consented to participate in the study, resulting in a response rate of 4.9%. In the following, we summarize a selection of original replies including English translations of the participants in Table 1, full replies can be found in Supplementary Appendix C (original in German) and Supplementary Appendix D (English translation).

**TABLE 1:** Selection of original transcribed interview answers in German and English translations.

Topic	English	German (original)
Definition of placebo	“There, the question arises about these extended placebo definitions. There, one must also comment on it once more. And I believe the beginning is definitely to find a reasonable definition. What is generally considered a placebo by the medical profession or even by those working in the healthcare sector. That would already be very helpful or the first step towards it.” Participant 4	“Dort ist dann eben die Frage nach diesen erweiterten Placebodefinitionen. dort muss man sich auch noch einmal dazu äussern. Und ich glaube der Anfang ist sicher mal, eine vernünftige Definition zu finden. Was man überhaupt als Ärzteschaft oder sogar als im Gesundheitswesen Tätiger als Placebo ansieht. Das wäre schon mal sehr hilfreich oder der erste Schritt dazu.” Participant 4
	“Many patients are already taking substances that don’t work. So, in those cases, the effect doesn’t come from an active ingredient, but simply from the tablet itself, the capsule, the powder. Then one doesn’t speak of placebo, those are some homeopathic remedies or herbal preparations. Vitamin tablets from Migros. This is not called a placebo, even though it has no proven effect.” Participant 1	“Etliche Patienten nehmen ja schon Mittel, die nicht wirken. Also bei denen die Wirkung nicht darin besteht, dass ein Inhaltsstoff darin wirkt, sondern es ist einfach die Tablette selbst, die Kapsel, das Pülverchen. Da spricht man dann eigentlich nicht von Placebo, das sind dann irgendwelche Homöopathischen Präparate oder pflanzliche Präparate. Vitamin-Tabletten aus der Migros. Dem sagt man nicht Placebo, obwohl es keine nachgewiesene Wirkung hat.” Participant 1
Use of placebos in clinical practice	“It’s not like I’m lying to the patient. With the sugar pill, I lie to him.” Participant 8	“Ich lüge ja den Patienten nicht an. Bei der Zuckerpille lüge ich ihn an.” Participant 8
Support for placebo administration	“Magnesium is also something that does people a lot of good. Then you cheat your way through a bit.” Participant 9	“Auch Magnesium ist was, das den Leuten sehr guttut. Dann mogelt man sich ein bisschen so durch.” Participant 9
	“I occasionally have NaCl injected. It’s a placebo. In this case. But it works. And of course, it’s about her (a patient) having a contact and not being so uptight. I’m doing something harmless. It would be much worse if I kept increasing the morphine until I basically medicate them to death. I find that difficult. Because she doesn’t have tumor pain. If it were a real tumor pain, one would say, we are initiating the final stage. Up with morphine, until respiratory depression. But she’s not in the stage. That’s why I don’t want to kill her. There, that’s a solution.” Participant 8	“Da lasse ich zwischendurch einmal NaCl spritzen. Ist ein Placebo. In diesem Fall. Aber er wirkt. Und es geht natürlich darum, sie hat dann einen Kontakt und sie ist nicht so vernagelt. Ich mache ja etwas Unschädliches. Es wäre viel schlimmer, ich würde im Morphium immer weiter hoch gehen, bis ich sie quasi zu Tode mediziere. Da habe ich Mühe. Denn sie hat keinen Tumorschmerz. Wenn es ein echter Tumorschmerz wäre, dann würde man sagen, man leitet das Finalstadium ein. Rauf mit dem Morphium, bis zur Atemdepression. Aber die ist nicht im Stadium. Deswegen, ich will sie nicht töten. Da ist das eine Lösung.” Participant 8
	“Yes, it’s basically no harm. You kind of deceive the patient a little. But often, when the course is already heading towards improvement, you just reassure them and they think ‘oh, he is taking good care of me.’ And he is not getting worse. On the contrary.” Participant 6	“Ja, das ist im Prinzip kein Schaden. Man bescheisst den Patienten ein wenig. Aber oft, wenn der Verlauf eh in Richtung Besserung ist, hat man einfach vertröstet und er denkt: ‘doch der schaut gut.’ Und es geht ihm ja nicht schlechter. Im Gegenteil.” Participant 6
	“So I believe that often in such cases where a placebo is given, a good conversation could also be had and then it might have been resolved. There’s often a lack of time.” Participant 9	“Also ich glaube, häufig in solchen Fällen, wo man Placebo gibt, könnte man auch ein gutes Gespräch machen und dann hätte sich das vielleicht erledigt. Da fehlt oft die Zeit.” Participant 9
	“Also if one is not sure whether a sedative is effective. But a sedative has side effects. It carries the risk of increased susceptibility to falls at night. The affected person can break their thigh, becomes immobile as a result, and might then get a pulmonary embolism and die. So, this is not something harmless. You don’t really know, does it work or doesn’t it? Then you can try to find out with placebos.” Participant 1	“Auch wenn man sich nicht sicher ist, ob ein Beruhigungsmittel etwas bringt. Aber ein Beruhigungsmittel hat Nebenwirkungen. Es hat das Risiko von vermehrter Sturzanfälligkeit in der Nacht. Der Betroffene kann sich den Oberschenkel brechen, ist von dem dann immobil und bekommt dann vielleicht eine Lungenembolie und stirbt. Also das ist nicht etwas Harmloses. Man weiss nicht richtig, wirkt es oder wirkt es nicht. Dann kann man versuchen, das mit Placebos herauszufinden.” Participant 1
Disapproval for placebo administration	“Especially in today’s times, when one deals with patients in a… patient-centered manner. I don’t believe that this is a basis for being able to deal with a patient in any meaningful long-term way if they eventually find out that they have been betrayed.” Participant 4	“Gerade in der heutigen Zeit, in der man. patienten-zentriert umgeht. Ich glaube nicht, dass das eine Basis ist, mit einem Patienten irgendwie sinnvoll langfristig umgehen zu können, wenn er dann irgendwann erfährt, dass man ihn hintergangen hat.” Participant 4
	“That one treats them properly and takes them seriously. And when one reaches their own limits, that one refers them to another specialist, logically. Because otherwise, they don’t have access to the healthcare system in my eyes.” Participant 4	“Dass man ihn anständig behandelt und ernst nimmt. Und wenn man selbst irgendwo anstösst, dass man dann weiter verweist, logischerweise. Weil sonst hat er nicht den Zugang zum Gesundheitswesen in meinen Augen.” Participant 4
Precondition	“You have to weigh everything in terms of benefits and risks. And if this ratio turns out to be positive, then it is acceptable. And if this benefit-risk ratio is acceptable, then it can be done. Whether it’s a skin incision during knee arthroscopy, a sugar pill, or a cream that actually only contains gelatin. Then that’s okay.” Participant 3	“Man muss dann doch alles abwiegen nach Nutzen und Risiko. Und wenn dieses Verhältnis positiv ausfällt, dann ist das in Ordnung. Und wenn diese Nutzen-Risiko-Verhältnis in Ordnung ist, dann kann man das machen. Sei es nun ein Hautschnitt bei der Kniearthroskopie oder eine Zuckerpille oder eine Creme, wo eigentlich nur Gelatine drin ist. Dann ist das ok.” Participant 3
	“I explained to him that, in my opinion, it has no effect. The costs are not too high. Well then, let’s just do it. If you don’t expect any side effects or anything like that. For me, in this sense, it’s not a real placebo because he is informed about it. Of course, this is something where, according to conventional medical knowledge, I would say it has no effect. But that’s just being done in the Eastern Bloc now, and there they find that it has an effect. Well then. Then I can live with that. But something where I deceive the patient, saying I am giving them something that has an effect, but I know exactly that it has no effect. For me, that’s a different level.” Participant 4	“Ich habe ihn aufgeklärt, dass das aus meiner Sicht keine Wirkung hat. Die Kosten sind nicht allzu hoch. Ja nun, dann machen wir das halt. Wenn man keine Nebenwirkungen oder so erwartet. Das ist für mich in diesem Sinn kein wirkliches Placebo, weil er da eigentlich aufgeklärt ist. Klar ist das etwas, bei dem ich nach schulmedizinischem Wissen sage, das bringt keine Wirkung. Aber das wird jetzt halt im Ostblock gemacht und dort findet man, dass es eine Wirkung bringt. Tja dann. Dann kann ich damit leben. Aber etwas, bei dem ich dem Patienten etwas vorgaukle, sage, ich gebe ihm etwas, dass eine Wirkung hat, ich aber genau weiss, dass es keine Wirkung hat. Das ist dann für mich schon noch ein anderes Level.” Participant 4
	“I say very clearly, the evidence situation is like this: There are people who say it helps them. But there are also other people who say it doesn’t help. We’ll try it out for a while. Three, six months, then we’ll drop it and then you tell me in the end if it helped you. Then we can continue. It didn’t help, so let’s leave it out. That’s how I do it, for example, with the preparation that almost leans a bit towards placebo. That’s how you could do it, by playing with open cards. Say, okay, there is no clear evidence for it, but we can try it anyway.” Participant 3	“Da sage ich ganz klar, die Studienlage ist so. Es gibt Leute, die sagen, es hilft ihnen. Es gibt aber auch andere Leute, die sagen es hilft nicht. Wir probieren das mal aus für eine Zeit. Drei, sechs Monate, dann lassen wir es weg und dann sagen sie mir am Ende, hat es ihnen etwas gebracht. Dann können wir weiter machen. Es hat nichts gebracht, dann lassen wir es weg. So mache ich das zum Beispiel mit dem Präparat, dass schon fast so ein bisschen in die Richtung Placebo geht. So könnte man das machen, in dem man tatsächlich mit offenen Karten spielt. Sagen, ok es gibt keine klare Evidenz dafür, aber probieren können wir es trotzdem.” Participant 3
Source of information and personal knowledge on placebo regulation	“But from there, I believe I know that it is already something that is difficult with the legal circumstances. Because it is essentially a deliberate deception. And if there is no consent, then it is not consent, so I believe legally it is not just a gray area, but rather not allowed, as I understand it.” Participant 7	“Aber von dort meine ich zu wissen, dass es schon etwas ist, das schwierig ist mit den gesetzlichen Gegebenheiten. Weil es im Prinzip ein bewusstes Irreführen ist. Und wenn kein Einverständnis vorliegt, dann ist es kein Consent also ich glaube rechtlich ist es eben eigentlich nicht nur eine Grauzone, sondern eher nicht erlaubt, so wie ich es verstehe.” Participant 7
Perspective on regulations		
Arguments in favor of specific regulations	“I believe in legal matters, it would be important to me to have a position that one is not considered a fraudster.” Participant 8	“Ich glaube im Rechtlichen wäre mir wichtig, eine Stellung, dass man nicht als Betrüger gilt.” Participant 8
	“It is indeed important that there is such an instance that one can align with if needed. I simply don’t have that need when it comes to placebo. But others might have that. And then it would be good if they had something. But one has to know that this evidence is reliable.” Participant 1	“Es ist schon wichtig, dass es eine solche Instanz gibt, nach der man sich ausrichten kann bei Bedarf. Ich habe einfach diesen Bedarf nicht, was Placebo anbelangt. Aber andere haben den vielleicht. Und dann wäre es gut, wenn sie etwas haben. Aber man muss wissen, das ist verlässlich, diese Evidenz.” Participant 1
	“So if this is really a big issue, that placebos are being used unlawfully, but patients have no way to easily defend themselves. There, of course, a regulation would help. But from my perspective, the first thing to clarify is whether it is really a problem.” Participant 7	“Also wenn das jetzt wirklich ein grosses Thema ist, dass Placebos widerrechtlich eingesetzt werden, aber Patienten da gar nicht die Möglichkeit haben, sich auf eine einfache Art zu wehren. Dort würde dann natürlich eine Regulation natürlich helfen. Aber dort wäre aus meiner Sicht zuerst einmal zu klären, ist es wirklich ein Problem.” Participant 7
	“So in this sense, it is also a non-therapeutic agent that is then administered under medical supervision or medical care. And therefore, it must be regulated. [who should regulate this?] Swissmedic. Central authority that oversees all medications, whether they contain active ingredients or not.” Participant 3	“Also in diesem Sinne ist das ja auch ein Nicht-Therapeutikum, das dann ja unter ärztlicher Leistung oder ärztlicher Aufsicht abgegeben wird. Und von daher muss das reglementiert werden. [who should regulate this?] Swissmedic. Zentrales Organ, das für alle Arzneimittel, sei es nun mit oder ohne Wirkstoff, die Aufsicht hat.” Participant 3
Arguments against specific regulations	“There, they can create the deterrence scenario, as is done, for example, with these banking regulations with these enormous fines. But there it also shows that these fines are needed again and again that it doesn’t really work.” Participant 4	“Da können sie das Abschreckungsszenario machen, wie das zum Beispiel mit diesen Bankenregularien gemacht wird mit diesen enorm hohen Bussen. Aber dort zeigt es sich ja auch, dass es immer wieder diese Bussen braucht und dass es nicht wirklich funktioniert.” Participant 4
	“I would very much like a discussion to be held about such things and for it to be documented in writing, but one must be aware that ethical principles are also subject to change. That is not a fixed thing.” Participant 1	“Ich wünschte mir sehr, dass man eine Diskussion über solche Dinge führt und dass man das auch schriftlich festhält, aber man muss sich bewusst sein darüber, dass auch ethische Grundsätze einem Wandel unterworfen werden. Das ist nichts Fixes.” Participant 1

### 3.1 Definition of placebo

Participants defined placebos as dummy drugs lacking active ingredients, noting that placebos can include not just pills but also injections, creams, or specific exercises. Some argue that every doctor–patient interaction involves a form of placebo, as therapeutic conversation itself can have a healing effect without any specific substance. Several participants mentioned that deception is a component of placebo interventions. During the interviews, the participants articulated their own interpretations of what constitutes a placebo, highlighting the absence of a universally accepted definition. When discussing impure placebos, participants either argued that these are not true placebos or struggled to define what qualifies as an impure placebo. They found it challenging to delineate where placebo interventions begin and end.

The attitudes of participants regarding the use of placebos in clinical practice can be categorized into three positions: those who support it, those who disapprove, and those who consider it acceptable under certain conditions (see Table 1 for summary). Only a few participants held unequivocal positions either in favor of or against the use of placebos. Most participants believe that specific circumstances and conditions need to be met for them to endorse placebo administration. When these conditions were satisfied, they view placebos as valuable tools in their practice. A careful evaluation of the risks and benefits associated with placebo use was deemed essential by all participants. The subsequent sections will outline the arguments for each of the three positions. Additionally, the distinction between pure and impure placebos was significant for most participants. Some viewed pure placebos were as unethical, while impure placebos were considered less harmful and were generally prescribed without moral reservations. Impure placebos were generally reported to be used more frequently, while references to pure placebos were less common. Furthermore, all participants appeared open to discussing their experiences with placebo administration.

### 3.2 Support for placebo administration

Participants frequently cited effectiveness as an argument for placebo administration, often linked to the notion that effectiveness is a crucial factor in any treatment. Participants shared instances where they successfully utilized placebos. Participants also noted using placebos when they believe the placebo poses less risk than medications with specific ingredients, particularly saline injections instead of opioids. In these cases, harm reduction was a priority, as they assessed that using a placebo was less damaging than increasing opioid dosages.

Some participants rationalize placebo use by suggesting that deception is not overly problematic. One noted that while placebos involve some betrayal, their effectiveness mitigates any harm. Another participant stated that if the placebo is effective, there is no real betrayal. Additionally, participants reported using impure placebos when patients persistently requested medication, reflecting patients’ perceptions of effective treatment. Some participants informed patients about the limited evidence for certain treatments, while others administered impure placebos without detailed explanations, sometimes exaggerating the treatment’s effectiveness. Time pressure also influenced placebo use, as some participants felt that administering a placebo was quicker than having an in-depth conversation with the patient. Lastly, some participants mentioned using pure placebos for diagnostic purposes, with informed consent from the patient, primarily to evaluate the effectiveness of medications that have severe side effects.

### 3.3 Disapproval for placebo administration

Despite the prevalence of placebo use among participants, various arguments against their application were highlighted. Several participants contend that the use of placebos could jeopardize the doctor–patient relationship. Deceptive administration of placebos is perceived as a breach of trust, which can have lasting detrimental effects on the relationship. Others deem the use of placebos unnecessary, arguing that the symptoms treated with placebos could be addressed through other medications or interventions. One participant emphasized that it is a physician’s responsibility to ensure patients have access to the healthcare system, suggesting that primary care physicians should refer patients to specialists before resorting to placebos. A participant asserted that failing to do so denies the patient genuine access to healthcare.

One participant claimed that administering placebos without the patient’s informed consent is unjustifiable. In rare instances where patients pose a danger to themselves or others, informed consent may be waived; nevertheless, in other situations, consent is required, necessitating a complex procedure. This participant deems the omission of informed consent in placebo administration unjustifiable in comparison to such circumstances because the advantages of placebo administration are insufficient to deceive the patient. Regarding informed consent, three participants indicated that deceit is a significant argument against the use of placebos. For them, dishonesty contradicts ethical standards and should hence be eschewed. Additional reasons to dismiss placebo administration include the potential negative effects, particularly from impure placebos, that can never be eliminated entirely. Moreover, with impure placebos, it was contended that administering medications without a medical indication should unequivocally be regarded as medical misconduct.

### 3.4 Preconditions for placebo administration

Placebo administration is generally considered acceptable by participants only under certain conditions, primarily related to a perceived risk-benefit ratio. Participants believe that placebos can be justified when used with good intentions to enhance care and attention. However, they should not be prescribed merely to appease difficult patients or their relatives, nor should costly treatments lacking evidence of effectiveness be given to those who cannot afford them. The patient’s integrity and health must not be compromised, and a trusting relationship between the patient and doctor is essential. In assessing risk-benefit scenarios, there were instances where administering a placebo was seen as less harmful than other medications, particularly those with high addiction potential or severe side effects. In such cases, participants viewed placebos as a preferable option for the patient.

Some participants argue against using placebos when effective medications are available, while others believe they are justified as a last resort when all other treatments have failed. For example, one participant mentioned using vitamin C infusions for unexplained fatigue after ruling out organic and functional causes. Another participant emphasized that only authorized treatments should be used as impure placebos, as some medications, despite limited effectiveness, provide physicians with a sense of security. Documentation was highlighted as crucial for administering placebos, ensuring transparency and traceability. Most participants stressed the importance of informed consent, which includes informing patients when an intervention is supported by little or no evidence. Many would explain that, despite limited evidence, the treatment could still be considered. Some participants suggested that increasing patients’ expectations by exaggerating a treatment’s effectiveness might be necessary.

### 3.5 Source of information and personal knowledge on placebo regulation

The majority of participants indicated that they had not sought information on current regulations. Regarding placebo administration in clinical practice, while only two had attempted to find specific details on the subject. Some noted that the topic was addressed during their medical education or continuing education. When queried about their knowledge of regulations surrounding placebo use, responses varied significantly. Many participants vaguely referred to laws that could potentially restrict placebo administration, with most expressing the belief that such practices are generally not permitted in clinical settings. Some suggested that while there may not be explicit laws, the interpretation of existing regulations could lead to a prohibition of placebo use.

In addition to legal considerations, some participants cited ethical guidelines that conflict with placebo administration, highlighting issues such as patient autonomy, the obligation to inform patients about their treatment, and the element of deception. Many participants expressed uncertainties regarding regulations and acknowledged their lack of in-depth knowledge about the legal aspects of placebo use. To obtain legal advice or further information on placebo regulations, participants indicated they would reach out to various expert bodies. The juridical expert body of the Swiss Medical Association FMH was frequently mentioned, along with other potential sources of information including the Kantonales Heilmittelinstitut (Cantonal Agency for Therapeutic Products), Swissmedic (Swiss Agency for Therapeutic Products), Kantonsärzt*in (Cantonal Officer of Health), Kantonsapotheker*in (Cantonal Pharmacist), and ethicists employed at larger hospitals in the region. One participant observed that patients who are lawyers might act as valuable information resources, or that colleagues could be sought for support. In addition to consulting specialists, a frequently mentioned approach for acquiring information about legislation includes conducting your own research through online search engines or exploring databases for pertinent material.

### 3.6 Perspective on regulations

Participants were inquired about their receptiveness to proposed laws concerning the use of placebos in clinical practice. Overall, both people who advocate for placebo use and those who oppose it in clinical practice do not perceive a necessity for regulations. A minority of participants expressed strong support for specific regulations. Nonetheless, the motivations underlying the attitudes differed significantly. The subsequent parts will present the arguments and their underlying rationale to facilitate a clearer understanding of the perspectives expressed. Furthermore, it is noteworthy that nearly all participants would advocate for a differentiation between pure and impure placebos under regulatory frameworks.

### 3.7 Arguments for specific regulations

Arguments for specific regulations on placebo use in clinical practice were discussed, despite most participants feeling no need for them. Participants emphasized the importance of individual evaluation for each treatment and doctor–patient relationship, highlighting that the risk-benefit ratio is crucial in placebo administration. They argue that regulations should mandate individual assessments for each case. Those opposed to deception insist that placebos should only be used with full patient awareness and consent, advocating for shared decision-making to be included in any potential regulations. A few participants express a desire for specific regulations to legally support their use of placebos in certain situations.

The formation of a scientific working group to outline appropriate applications for placebos was suggested, which could provide clear guidelines for practitioners. One participant noted that even if placebo use is not common in their practice, regulations could assist others, proposing that the Swiss Medical Association FMH could develop consensus guidelines. Some participants argue that specific regulations should only be established if there is a significant number of lawsuits related to deceptive placebo use, suggesting that a legal framework could enhance patient rights. This perspective contrasts with the view that placebo administration is a medical service regulated by Swissmedic, the Swiss Agency for Therapeutic Products, which oversees the licensing and regulation of therapeutic products.

### 3.8 Arguments against specific regulations

Many participants expressed a general lack of confidence in medical regulations, regardless of their views on placebo use in clinical practice. Some argue that there are already too many regulations, while others highlighted the challenges of regulating medical consultations and placebo administration due to the uniqueness of each patient and physician, making it difficult to draw clear legal distinctions. Additionally, some participants feel that medical laws do not achieve the desired outcomes. In this context, several participants advocated for a discourse-based approach rather than strict regulations.

Participants who frequently use placebos expressed concerns that regulations could lead to a complete ban on their use, which they believe would restrict patient autonomy. They reason that patients should have the right to choose treatments without specific effectiveness, citing dietary supplements as an example of such treatments that can still provide relief based on patients’ beliefs. Some participants discussed individual risk-benefit assessments in relation to regulations, with opinions divided on whether this supported or opposed specific regulations. They emphasized the need for flexibility and individuality in treatment, arguing that existing legal and ethical frameworks provide sufficient guidance for determining the justification of placebo administration. Those opposed to placebo use contended that regulations would be irrelevant to their practice.

Even though nearly all participants use at least impure placebos, many stated that no additional regulations are necessary, as current laws and ethical principles already prohibit placebo administration. The need for informed consent was highlighted as a key ethical principle guiding placebo use. One participant expressed uncertainty about their knowledge of regulations, noting that this lack of clarity provides a sense of safety, potentially exonerating the physician in legal situations.

## 4 Discussion

This qualitative study explored attitudes of 10 Swiss primary care physicians toward placebo regulations in clinical practice. The findings reveal nuanced perspectives shaped by clinical experience, ethical considerations, and skepticism toward formal regulations. Participants broadly defined placebos as dummy treatments, such as pills or injections lacking specific efficacy. While some of the participants acknowledged the role of the doctor–patient relationship in placebo use, only a few participants explicitly identified deception as an essential component, with little mention of the emerging concept of open-label placebos. A lack of familiarity with impure placebos was evident among the 10 participants, consistent with findings from Linde et al.’s review (7). This ambiguity reflects ongoing scientific debates surrounding the definition and classification of placebos, particularly impure ones, which have been critiqued as “unsound and absurd” in previous works (7, 14, 22, 23). Furthermore, this knowledge gap points to the need for clearer communication and education about placebo classifications within the medical community. Participants exhibited varied ethical stances on placebo use, with decisions heavily influenced by risk-benefit analyses and their personal beliefs, echoing findings from prior research (11). The subjective nature of these assessments’ hints at lack of consistency and objectivity in clinical decision-making.

Many participating physicians adopted an approach of prioritizing perceived patient benefit over strict respect for autonomy. While beneficence served as a guiding principle, this approach often bypassed shared decision-making, leaving gaps in patient involvement and autonomy. Informed consent was typically sought, yet some participants exaggerated treatment efficacy to bolster placebo effects, a practice that raises ethical concerns. This understanding aligns with the study’s findings of minimal ethical discomfort around deception, as participants believed patients rarely discovered they had been misled.

The complexity of Swiss medical law (12) and participants’ general mistrust of regulations further shaped their attitudes. While most physicians expressed uncertainty regarding existing legal frameworks for placebo use, they rarely sought guidance from legal or expert bodies. Institutions such as the Swiss Medical Association (FMH) were seen as potential sources of information, but participants reported a lack of specific and actionable guidelines. This gap points to potential uncertainties within the system and suggests the value of developing clearer frameworks that consider the ethical, clinical, and legal dimensions of placebo use.

Notably, most participants opposed formal regulations for placebo administration, diverging from quantitative studies that advocate for specific guidelines (11, 14). Their opposition stemmed from a deep skepticism toward regulatory systems, driven by concerns that formalized rules might restrict clinical flexibility and disrupt the therapeutic application of placebos. This perception is particularly relevant for impure placebos, which were widely used despite confusion surrounding their definition and ethical implications. The findings suggest that while physicians are aware of ethical dilemmas associated with deception, their reliance on paternalistic decision-making forwards a belief that beneficence outweighs concerns about autonomy. This perspective contrasts with evidence that patients often accept placebo effects when transparency is maintained (24–27). Studies have shown that openly administered placebos can be effective in both clinical and non-clinical settings, providing a compelling alternative that balances therapeutic benefit with respect for patient autonomy (17).

To advance the discussion, future research should explore the perspectives of patients, as their preferences and expectations are central to resolving the tension between beneficence and autonomy. Relevant institutions and bodies, like medical associations, patient groups, scientists and regulatory authorities, could play a pivotal role in developing evidence-based guidance that supports ethical and effective placebo administration (see for example Evers et al. (28)). Addressing these challenges will foster a more transparent and ethically sound integration of placebos in clinical practice while maintaining their therapeutic potential.

### 4.1 Limitations

This study has several limitations. Given its qualitative nature, the findings are context-specific and may not be transferable to all physicians in Switzerland. The purposive sampling strategy, combined with recruitment from only two German-speaking cantons, means that the perspectives captured are shaped by the local professional and cultural context. Rather than seeking statistical generalizability, this study aims to provide in-depth insights into the views of a particular subset of physicians (29, 30).

Additionally, our sample was highly selective due to self-selection bias. With a response rate of 4.9%, it is likely that those who chose to participate had a particular interest in or experience with placebo use. The interviews suggest that participating physicians were primarily those who actively use placebos, while others who declined participation may either not use placebos, not perceive the topic as relevant, or not recognize their use of impure placebos. This may have influenced the range of perspectives captured. Furthermore, social desirability bias cannot be ruled out, as participants may have framed their responses in ways they deemed professionally or ethically appropriate. Recruiting primary care physicians for qualitative interview studies is known to be challenging due to time constraints and clinical workload, and no compensation was offered in this study. These factors likely contributed to the low response rate, while a limitation, is common in interview-based research targeting this population.

Despite these limitations, the 10 interviews provide rich, foundational insights into physician perspectives on placebo use in the German-speaking regions of Switzerland (31). Future research should seek to include participants from additional Swiss regions, ensure greater diversity in gender and professional backgrounds, and incorporate the perspectives of patients to further explore the balance between beneficence and autonomy in clinical practice.

## 5 Conclusion

This study illustrates that primary care physicians recognize the therapeutic value of placebos, particularly impure ones, but navigate a complex ethical landscape where beneficence and autonomy are often in tension. The investigated subset of physicians adopted an approach to placebo use which prioritized perceived patient benefit over strict adherence to autonomy, yet remain skeptical of formal regulations, fearing they might restrict clinical flexibility and limit the therapeutic potential of placebos. Despite the selective sample and the small sample size, these findings suggest the need for more discussions on placebo use, integrating patient perspectives to address ethical concerns and advance the understanding of its role in modern clinical practice.

## Data Availability

The original contributions presented in this study are included in this article/Supplementary material, further inquiries can be directed to the corresponding author.
