# Supplementary material for: A dose of doubt: a qualitative study on placebo regulations
Source: Front Med (Lausanne). 2025 Jun 16;12:1574022. doi: 10.3389/fmed.2025.1574022 (PMC12206773; doi:10.3389/fmed.2025.1574022)
Supplement: Supplementary file 1 [file Supplementary_file_1.docx]

Supplementary Material

# Appendix A. Interview Guide Original and English Translation

**Thema und Leitfrage | Nachfragen**

**Definition**

- **Wie definieren Sie ein Placebo?**
  - Bitte beschreiben Sie, was Sie unter einem Placebo verstehen.

**Anwendung in der medizinischen Praxis**

- **Wie findet der Einsatz von Placebos Eingang in Ihre berufliche Praxis?**
  - Wo begegnen Ihnen Placebos in Ihrem Arbeitsalltag?
  - Wie stehen Sie zum praktischen Einsatz von Placebos?

**Informationsbeschaffung**

- **Wie beschaffen Sie sich Informationen über geltende Regelungen und Richtlinien zum Placeboeinsatz?**
  - Haben Sie sich schon einmal über die rechtlichen Regelungen zu Placebos informiert?
    - Wenn ja, wie sind Sie vorgegangen/welche Kanäle haben Sie genutzt?
    - Wenn nein, wie würden Sie vorgehen?
  - Sind Ihnen Regelungen bekannt, die sich auf den Placeboeinsatz beziehen?

**Art der Regelung**

- **Welche Art von Regelung zum Placeboeinsatz finden Sie angemessen?**
  - Wie detailliert sollten diese Regelungen sein?
  - Sind in bestimmten Bereichen stärkere Regelungen notwendig? Wenn ja, in welchen?
  - Welche Inhalte sollten in diesen Regelungen enthalten sein?
  - Auf welcher Ebene sollten diese Regelungen angesiedelt sein? (Statements, Empfehlungen, Berufskodizes oder Gesetze)
  - Wie verbindlich sollten diese Regelungen sein?
  - Macht es für Sie einen Unterschied, ob es sich um ein „reines“ Placebo handelt (z. B. Zuckerpillen) oder um ein „unreines“ Placebo (z. B. Antibiotika bei viralen Infekten, Behandlungen ohne nachgewiesene Wirkung, die aber aufgrund der Hoffnung und Erwartungen der Patient:innen wirken)?
    - Bitte erklären Sie Ihre Antwort.

**English Translation:**

**Topic and Guiding Question | Follow-Up Questions**

**Definition**

- **How do you define a placebo?**
  - Please describe what you understand by a placebo.

**Application in Medical Practice**

- **How is the use of placebos integrated into your professional routine?**
  - Where do you encounter placebos in your daily work?
  - What is your perspective on the practical use of placebos?

**Accessing Information**

- **How do you obtain information about the applicable regulations and guidelines for placebo use?**
  - Have you ever tried to inform yourself about the legal regulations surrounding placebos?
    - If yes, how did you proceed/which channels did you use?
    - If no, how would you approach this?
  - Are you aware of regulations that pertain to placebo use?

**Type of Regulations**

- **What kind of regulation for placebo use do you find appropriate?**
  - How detailed should these regulations be?
  - Are stronger regulations needed in certain areas? If so, which ones?
  - What content should be included in these regulations?
  - At what level should these regulations be established? (Statements, recommendations, professional codes, or laws)
  - How binding should these regulations be?
  - Does it make a difference to you whether the placebo is a "pure" placebo (e.g., sugar pills) or an "impure" one (e.g., antibiotics for viral infections, treatments with no proven effect but effective due to patient hope and expectations)?
    - Please explain your answer.

# Appendix B: Coding System

| **Main categories** | **Subcategory (1)** | **Subcategory (2)** | **Subcategory (3)** | **Subcategory (4)** |
| --- | --- | --- | --- | --- |
| Definition of placebos | Therapeutic agent | No consistent definition |  |  |
| Placebo use in clinical practice | Attitude towards Placebo administration | Application situation |  |  |
| Information retrieval | Known regulations | Assessment of own knowledge | Information attempts | Potential sources of information |
| Attitude towards regulations | No need for regulations | No need for regulations |  |  |
